# Structural reorganisation of cellulose fibrils in hydrothermally deconstructed lignocellulosic biomass and relationships with enzyme digestibility

**DOI:** 10.1186/1754-6834-6-33

**Published:** 2013-03-02

**Authors:** Roger Ibbett, Sanyasi Gaddipati, Sandra Hill, Greg Tucker

**Affiliations:** 1 BBSRC Sustainable Bioenergy Research Centre, University of Nottingham, Sutton Bonington, Leicestershire, Loughborough, LE12 5RD, UK

**Keywords:** Biomass, Fibril, Morphology, Accessibility, Reactivity, Enzyme, Hydrothermal, Pre-treatment

## Abstract

**Background:**

The investigation of structural organisation in lignocellulose materials is important to understand changes in cellulase accessibility and reactivity resulting from hydrothermal deconstruction, to allow development of strategies to maximise bioethanol process efficiencies. To achieve progress, wheat straw lignocellulose and comparative model wood cellulose were characterised following increasing severity of hydrothermal treatment. Powder and fibre wide-angle X-ray diffraction techniques were employed (WAXD), complemented by enzyme kinetic measurements up to high conversion.

**Results:**

Evidence from WAXD indicated that cellulose fibrils are not perfectly crystalline. A reduction in fibril crystallinity occurred due to hydrothermal treatment, although dimensional and orientational data showed that fibril coherency and alignment were largely retained. The hypothetical inter-fibril spacing created by hydrothermal deconstruction of straw was calculated to be insufficient for complete access by cellulases, although total digestion of cellulose in both treated straw and model pulp was observed. Both treated straw and model pulps were subjected to wet mechanical attrition, which caused separation of smaller fibril aggregates and fragments, significantly increasing enzyme hydrolysis rate. No evidence from WAXD measurements was found for preferential hydrolysis of non-crystalline cellulose at intermediate extent of digestion, for both wood pulp and hydrothermally treated straw.

**Conclusions:**

The increased efficiency of enzyme digestion of cellulose in the lignocellulosic cell wall following hydrothermal treatment is a consequence of the improved fibril accessibility due to the loss of hemicellulose and disruption of lignin. However, incomplete accessibility of cellulase at the internal surfaces of fibrillar aggregates implies that etching type mechanisms will be important in achieving complete hydrolysis. The reduction in crystalline perfection following hydrothermal treatment may lead to an increase in fibril reactivity, which could amplify the overall improvement in rate of digestion due to accessibility gains. The lack of preferential digestion of non-crystalline cellulose is consistent with the existence of localised conformational disorder, at surfaces and defects, according to proposed semicrystalline fibril models. Cellulases may not interact in a fully selective manner with such disordered environments, so fibril reactivity may be considered as a function of average conformational states.

## Background

Physico-chemical deconstruction is important as a means of increasing the efficiency of enzyme hydrolysis of cellulose in the lignocellulosic cell wall, in order to liberate fermentable sugars for production of ethanol. The cellulose fibrils in the native cell wall consist of crystalline assemblies of polymer chains, constrained by highly organised Van-der-Waals and hydrogen bonding. Despite the inherent hydrophilicity of the molecular chains, this organised structure is impenetrable to water and cannot be swelled or dissolved by common solvents. Enzyme hydrolysis reactions in the native cell wall are therefore restricted to the exterior fibril surfaces, or the limited defects which may exist along fibril lengths [1]. In addition, the elementary fibrils are sheaved in a layer of hemicellulose polymer, which acts as an interface with the surrounding lignin matrix [2]. This complex composite must therefore be at least partly deconstructed in order to increase the accessibility and reactivity of the cellulose polymer.

Various physico-chemical deconstruction treatments have been developed for bioethanol production, which operate by differing mechanisms on different components in the cell wall [3]. Some are effective by chemically removing all or part of the lignin fraction, for example by extraction using hot aqueous alkali [4] or by organosolv techniques [5]. Other approaches are based on the physical solvation or swelling of the cellulose and hemicellulose fractions, for example using ionic liquids [6]. One of the most promising deconstruction methods for lignocellulosic biomass makes use of hydrothermal conditions, where the cell wall is disrupted by reaction with water at high temperature and pressure, which causes hydrolysis of the hemicellulose component and degradation followed by re-condensation of the lignin component [7]. Such hydrothermal methods have low chemical demand and result in a deconstructed product with high enzyme digestibility, which are key benefits for successful operation at industrial scale. However, challenges still need to be overcome before hydrothermal processing becomes a commercial reality, including the management of reaction biproducts such as aromatic, furanic and organic acid compounds, which inhibit fermentation [8]. Also, the need for operation at high temperatures leads to a high energy demand, with consequent impact on the overall environmental balance, also leading to engineering challenges in reactor design for containment of water at temperature and pressures.

A better understanding of the mechanisms of hydrothermal deconstruction of lignocellulosic biomass is required, in order to establish process conditions which minimise production of unwanted biproducts, whilst maximising enzyme digestibility of the treated product, also minimising energy consumption. In this respect it is important to understand the morphological changes that take place within the lignocellulose cell wall as a result of deconstruction, to identify the critical structural factors which influence the rate and extent of enzymatic saccharification, and to investigate how such changes may be optimised for highest saccharification efficiency [9,10]. In particular, there is uncertainty surrounding the potential structural reorganisation of cellulose fibrils during hydrothermal treatment and its significance in relation to digestibility, which has been the focus for this study. Firstly, the study has been aimed at gaining a fuller understanding of fibril organisation in the cell walls of wheat straw, as a model for lignocellulose, by use of wide angle X-ray diffraction (WAXD) and supporting techniques. The study has then systematically followed changes in fibril organisation caused by hydrothermal treatment of straw under controlled conditions of increasing severity. The impact of fibril structural reorganisation on digestibility has then been interpreted by the application of a morphologically based kinetic model for enzyme hydrolysis [11].

Cellulose crystallinity has been explored as a variable with a potential impact on enzyme digestibility [1], but greater consideration needs to be given to the size, alignment and packing of cellulose fibrils within the cell wall matrix [12,13], as well as the co-organisation with hemicellulose and lignin [14]. Comparisons and contrasts with the properties and behaviour of a model high α-cellulose wood-pulp have formed an integral part of this study, together with comparisons of pulp and straw materials subjected to micro-fragmentation by high-shear wet attrition. To gain the most informative structural insights it is necessary to obtain quantitative crystallinity data from X-ray diffraction techniques, which in the context of cellulose and lignocellulose materials has been the subject of informative fundamental and comparative investigations [15-19]. For this current study, the merits of different protocols for WAXD crystallinity analysis were considered, with the aim of selecting a pragmatic, robust procedure, which could be applied with physical and morphological relevance to understand fibril and cell wall structural modifications.

## Results and discussion

### Cellulose fibril organisation in straw

**Figure 1:**
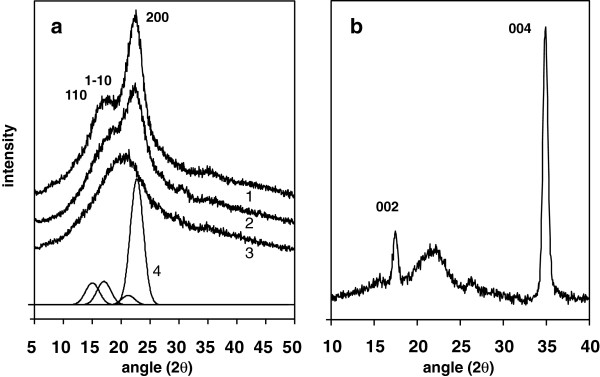
**(a) 2(**θ**) WAXD patterns of wheat straw by powder reflection method (offset for clarity): (1) untreated sample, (2) intermediate ballmilled sample, (3) highly ballmilled sample used as amorphous profile, (4) Gaussian fit of crystalline peaks from untreated sample; b) 2(θ) WAXD meridional pattern of aligned stem of untreated straw.**

The powder WAXD diffractogram of whole untreated straw showed the major crystalline peaks from the native cellulose-I allomorph from the fibrils of the cell wall, superimposed on a broader non-crystalline profile, as shown in Figure 1a. Differentiation between 1α and 1β sub-allomorphs was not possible due to the breadth of reflections, although it is anticipated that both straw and wood will consist mainly of the 1β structure [20]. The hemicellulose and lignin fractions of the cell wall contribute only to the non-crystalline profile, as these structural components have low stereoregularity and are unable to organise into repeating crystal structures. The study has made advantageous use of ballmilling techniques for controlled decrystallisation of lignocellulose materials, which directed analysis towards the use of amorphous subtraction and integration procedures (see Methods) [15]. Intensity and background correction procedures have been applied following the rationale of earlier published studies [15-17,19]. Figure 1a illustrates the progressive loss of the cellulose crystalline peaks with increasing ballmilling time, leading to the generation of a purely amorphous profile, which was subtracted for crystallinity integrations (equation 3, Methods). The effect of compositional differences on the amorphous profile shape was accounted for by using sample-specific amorphous curves. The change in amorphous coherent intensity with composition was not regarded as significant for hydrothermal treatment of similar biomass type [19]. The correction factor K in equation (3) accounts for the loss of crystalline intensity due to crystalline imperfections, which depends on the Ruland-Vonk disorder parameter k in equation (4). Literature values of k for cellulose of 2.5 Å^2^ have been used for all straw materials [21], and 1.8 Å^2^ for all eucalyptus materials [22]. No correction to equation (3) has been made for partitioning of residual moisture, as there is uncertainty about the exact morphological environment of water molecules, which according to deuteration studies may be at least partly accessible to crystallite surface layers and defects [23].

The inherent crystallinity of the cellulose fibril fraction of the straw cell wall was calculated from the total WAXD crystallinity, by reference to the glucan sugar composition established by total acid hydrolysis (see Methods). The assumption is that all glucose analysed in the straw is derived from cellulose, as previous studies have indicated that less than 1% of the total glucose is present in the hemicellulose fraction [24]. From this calculation the cellulose fibrils in untreated straw were estimated to be of 63 % crystallinity, which slightly lower than the 66% crystallinity estimated for the as-received eucalyptus pulp, in Table 1. Although various approximations have been made with the WAXD analysis, it is apparent that the fibrils in native straw are not perfectly crystalline, so must contain polymer chains in disordered environments, with imperfect inter-chain bonding. Such disorder is established in refined cellulose materials, where decrystallisation could be a biproduct of extraction procedures, but in the native state it must be the product of biogenesis and assembly of the cell wall. Higher crystallinity values for cellulose in plant materials have been determined using Rietveld refinement methods, which include intensities from unresolved minor reflections over the full 2θ range. However, values in excess of 100% have been quoted, possibly a result of uncertainty in the choice of crystalline peak shapes [19].

**Table 1:** Compositional and X-ray data for as-received and hydrothermally processed wheat straw and eucalyptus pulp, and partially enzyme digested materials

**Material**	**Process conditions**	**Compositional data (stem + leaf)**			**Powder WAXD data**			**Fibre WAXD data (stem internode)**			
	**hydrothermal reactor temperature (°C)**	**weight loss (wt-%)**	**glucan (wt-%)**	**xylan (wt-%)**	**measured crystallinity (%)**	**fibril crystallinity (%)**	**crystal width (200) (nm)**	**crystal length (004) (nm)**	**disorientation**		
									**(004) (FWHH°)**	**(200) (FWHH°) narrow broad**	
Wheat straw	as received	0	39.0	23.0	24.5	63	2.8	12.1	15.5	17.5	71
	155	10	43.5	24.2	29.2	67	3.0				
	180	14	47.4	24.1	28.6	60	3.2	11.8	14.7	19.6	108
	190	32	56.5	13.8	31.7	56	3.3				
	200	40	60.0	1.8	26.1	44	3.4	11.6	15.0	20.0	112
Eucalyptus pulp	as-received	0	96.0	4.0	63.6	66	3.6				
	180	4	96.5	1.2	67.7	70	4.2				
	195	4	97.0	1.1	71.8	74	4.4				
	200	4	96.9	1.1	72.0	74	4.6				
	**time of enzyme hydrolysis (hours)**	**glucose yield**^**+ **^**(%)**	**remaining glucan (wt-%)**		**measured crystallinity (%)**	**fibril crystallinity (%)**	**crystal width (200) (nm)**				
Hydrothermally processed wheat straw (190^o^C)*	0	0	59.0		35.4	56	3.3				
	6	43	45.1		28.3	58	3.4				
As-received eucalyptus pulp*	0	0	96.0		60.0	69	3.6				
	6	30	97.0		60.5	68	3.6				

* separate experimental set; ^+^ from enzyme digestion, as proportion of yield from acid total hydrolysis.

**Figure 2:**
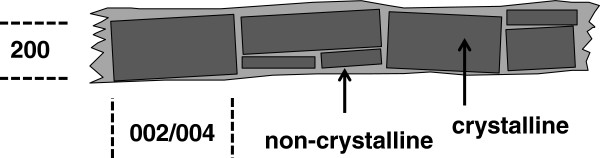
Schematic of elementary fibril of cellulose, showing WAXD reflections used to estimate lateral and longitudinal crystal dimensions.

The elementary cellulose fibril of the native cell wall may be represented as a connected string of crystalline domains, as illustrated in Figure 2, although this model does not preclude the assimilation of such elementary structures into larger micro-fibrils. If the crystalline organisation of the elementary fibrils is accepted, then the Scherrer calculation for the 200 reflection will give an indication of the average fibril cross-sectional dimension (equation 7, Methods). For the as-received straw this was found to be 2.8 nm, in Table 2, which was lower than the value of 3.6 nm for the eucalyptus pulp. No correction for broadening due to crystal defects was deemed necessary, due to the relatively large widths of the 200 peak, although the value will be imprecise due to a lack of certainty of sectional shape and the exact alignment of the 200 lattice in the fibrils [20,25]. In support of the model, for native cellulose fibrils it is known that the Scherrer lateral dimension follows a general upward trend with crystalline content, which is consistent with the organisation of non-crystalline cellulose chains as a fringe at fibril surfaces, resulting from their higher conformational freedom [26]. However, there is debate over the thickness of this fringe and the extent of disorder for required for polymer chains to be excluded from the inner crystalline domains of the fibril. A useful discussion of current thinking on fibril structure is found in reference [27], drawing from the results of X-ray, water sorption and solid state NMR studies. NMR measurements typically give rise to lower fibril crystallinities, as the conformational sensitivity of chemical shifts, especially as a result of C5-C6 rotation, means that a greater proportion of boundary chains and defects are included in the non-crystalline fraction.

**Table 2:** Kinetic parameters for enzyme hydrolysis of eucalyptus pulp and hydrothermally processed wheat straw

**Sample**	**Average normalised rate constant (min**^**-1**^**)**	**Fitted proportions (%)**			Fitted rate constants (*normalised by proportion) (min^**-1**^**)**		**R**^**2 **^**of fit**
		**Non digestible**	**Fast digesting**	**Slow digesting**	**Fast**	**Slow**	
As-received wheat straw	0.009	81	6	13	0.46 (*0.028)	0.058 (*0.008)	0.989
Straw hydrothermally processed to 180^o^C	0.03	30	17	53	0.65 (*0.11)	0.046 (*0.02)	0.996
Straw hydrothermally processed to 200^o^C	0.10	2	98	0	0.10		0.994
Neverdried straw hydrothermally processed to 200^o^C	0.14	1	99	0	0.14		0.993
As-received eucalyptus pulp	0.03	0	42	58	0.12 (*0.05)	0.020 (*0.01)	0.998
Fibrillated hydrothermally processed straw (200^o^C)	0.24	2	98	0	0.24		0.995
Fibrillated eucalyptus pulp	0.08	0	30	70	0.50 (*0.15)	0.065 (*0.05)	0.999

The geometrical fringe-to-crystal relations of the fringe-fibril model can be can be investigated by using equation 1 (from [23]). If the non-crystalline content determined by WAXD integration is 100-63 = 47%, giving the fractional value (A), and the dimension associated with crystalline diffraction (L) from the 200 Scherrer calculation for straw is 2.8 nm, then the apparent width of the fringe for straw is found to be 0.43 nm. There are significant limitations in the definitions of L, A and sectional shape, but in concept this calculation does suggest such a non-crystalline fringe might be one crystal layer or less in dimensions, and might be associated with other partially disordered surface chains with sufficient periodicity to be included in the crystalline fraction. A similar calculation for the eucalyptus pulp gives the apparent width of the fibril fringe as 0.48 nm. Additionally, assuming a total square cross-section and an average fibril density (ρ) of 1.55 g/cm^3^, then for straw the total fibril surface (S) from equation (2) would be 169 m^2^/g. The corresponding value for the eucalyptus pulp is 136 m^2^/g. In each case this represents the maximum theoretical area for enzyme interaction, although constraints due to inaccessibility of reactive centres or aggregation of elementary fibrils will reduce this, and in the case of straw this will also be significantly reduced by shielding from hemicellulose or lignin.

(1) $$h=\frac{L}{\left(4/A\right)-2}$$

(2) $$S=\frac{4.\rho }{L+2h}$$

An indication of the average crystal lengths within the fibrils was obtained by measurement of the integral breadth of the 002 and 004 reflection of cellulose-I, at 17.2° and 34.9° 2θ respectively. These are prominent features in the meridional diffractograms of the straw stems, illustrated in Figure 1b, due to the fibrils in the epidermal cells, which are preferentially oriented in the stem direction [28]. For these sharper peaks it is necessary to separate the contributions to peak broadening from finite crystal size and crystal defects, so the extrapolation procedure described in reference [27] (see Methods) was employed. The values determined by this procedure, around 12 nm for untreated straw in Table 1, recognise that breaks in crystalline domains exist along the fibril length, as in Figure 2, which could be susceptible to hydrolytic attack, by acid or enzyme action. A broad feature is also observed between 15 and 23° (2θ,) in Figure 1b, which is probably from fibrils in the inner parenchyma cells, with lower alignment with respect to the stem direction, but could also be from non-cellulose material.

**Figure 3:**
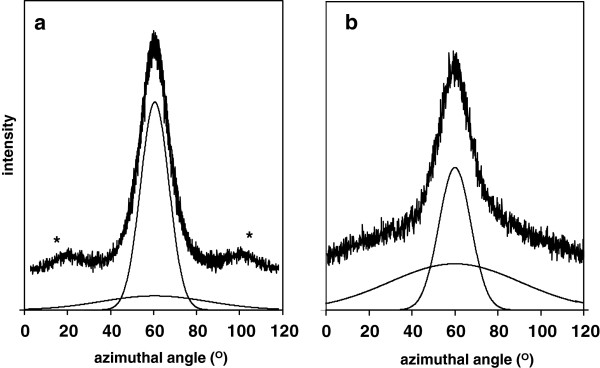
**Wide-angle X-diffraction scans of aligned stem of untreated wheat straw: (a) azimuthal scan at 2(θ) = 34.9° (004), (b) azimuthal scan at 2(θ) = 22.5° (200 + non crystalline material).** * = tails of off axis reflections.

The alignment of the fibrils with respect to the stem axis was determined from the distribution of azimuthal intensity of cellulose diffraction peaks of the stem sliver samples, described by fitting to one or more Gaussian functions [29,30]. An azimuthal scan at the cellulose-I 004 position (34.9° 2θ), in Figure 3a, revealed a sharp Gaussian profile from crystalline cellulose, centred on the equatorial line, which confirmed the high coherence of the fibrils of the epidermal cells. This suggests that the fibrils do not follow a helical path around the cell wall, as found for example with cotton. The full width at half-height (FWHH) of this profile was 15.5° corresponding to an average angle of disorientation ($\bar{\varphi }\;$) of 9.2°, (equation 5, Methods section), which may include contributions from the possible twist of crystal planes within the fibrils, which was not considered in this study. However, the effective fitting of the narrow 004 profile required the inclusion of a second broader profile, of lower intensity, also fitted to a Gaussian function, which was assigned to the less oriented cellulose fibrils from the parenchyma cells of the stem [28]. This component was absent from a comparable scan of a stem sliver from which the inner parenchyma material had been removed with a blade. For comparison, an equivalent azimuthal scan of an aligned leaf sample gave rise to an almost constant intensity at all angles (not shown), confirming that cellulose fibrils in the parenchyma leaf cells are almost randomly oriented. The wings marked with an asterix in Figure 3a are tails of off-axis reflections, possibly the 1-21 cellulose-I crystal planes.

An azimuthal scan of the stem sliver was also performed at the cellulose-I 200 position (22.5° 2θ), as shown in Figure 3b. The experimental profile could again be fitted as superimposition of a narrow and broad Gaussian functions, although the broad component accounted for much a larger proportion compared to the 004 scan, with a FWHH estimated at 71° and an average angle of disorientation ($\bar{\varphi }\;$) of 37°. The narrow component was again assigned to the oriented crystalline fibrils in the epidermal cells, and a proportion of the broad component was assumed to come from the less oriented parenchyma cells. However, the remainder of the broad component was interpreted as a superposition from other morphological entities, probably from non-crystalline cellulose but also from lignin and hemicellulose [31]. The diffraction from this partially aligned component will contribute to the non-crystalline intensity in the powder diffractograms, which will therefore be included in the respective crystallinity calculations. WAXD observation of orientation of non-crystalline domains is known from studies of synthetic polymers, where constructive interference occurs due to correlations between or within macromolecular chains, even without the requirement for full crystalline register [32]. Precise calculation of orientation parameters is difficult and is restricted in this paper, although in-depth studies will be possible in the future using 2D detector methods.

### Fibril reorganisation following hydrothermal deconstruction

**Figure 4:**
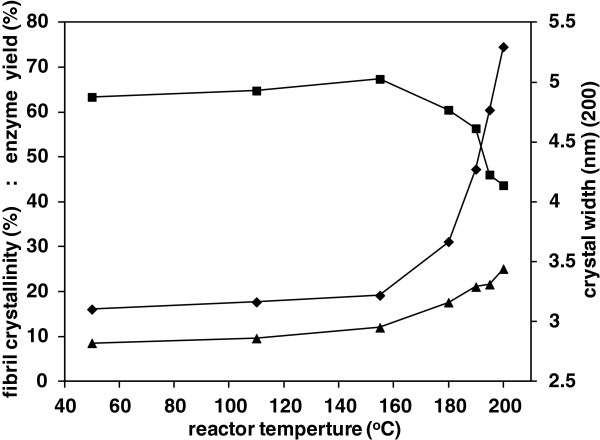
Variation in WAXD parameters and enzyme digestibility of straw treated at different temperatures; (■ ) fibril crystallinity, (♦ ) glucose sugar yield after 24 hr enzyme hydrolysis, (▲ ) fibril average width from fitted 200 reflection.

Precisional errors in the measurement of cellulose crystallinity come from a number of sources, with a standard deviation found to be 2.2 percentage units, based on a triplicate XRD and glucan analysis on the same treated straw material, with glucan values taken as an average of duplicates. With this consideration, the results suggest that the crystallinity of the fibrils in straw may have increased slightly following hydrothermal treatment at intermediate temperatures, in Figure 4, achieving a maximum around 67% for treatment at 155°C. However, this temperature is insufficient to induce the main hydrothermal reactions, with minimal increase seen in either extractable mass or enzyme digestibility. This small increase could therefore be due to physical annealing, aided by water plasticisation, which would be enhanced at elevated temperature. This involves the loss of crystal defects and strains and also possibly aggregation between smaller crystal domains, which is a phenomenon observed in both natural and regenerated cellulose material [33]. A slight increase in the apparent fibril width was seen, from 2.8 to 3.0 nm, in Table 1, which would support this interpretation.

The onset of the major hydrothermal reactions was evident as the reactor temperature exceeded 180°C, with both solubilised mass and enzyme digestibility increased significantly. Under these conditions hemicellulose is hydrolysed to xylose oligomers and monomers, which accounts for the majority of the solubilised material, with lignin becoming degraded and a minor proportion of lignin fragments also solubilised. The cellulose fibrils are not solublised at these temperatures, with a high proportion of glucan retained in the solid residue from the straw, from Table 1. However, the WAXD results in Table 1 show that some reorganisation may take place within the fibrils, with the calculated crystallinity progressively reduced from 67% to 44%, as the process temperature was increased from 155 to 200°C. This same phenomenon has recently been observed for sugarcane bagasse, where a Rietveld refinement was employed to calculate cellulose crystallinity values [19]. An increase in the paracrystalline content of hydrothermally treated switch grass has also been noted using solid state NMR [10]. The amorphous subtraction technique of the current study revealed that the cellulose-I 200 diffraction peak in straw became sharper at the highest hydrothermal reaction temperatures, indicating a further increase in apparent fibril width. This effect was also noted from the Reitvield analysis of hydrothermally treated bagasse. Possibly thinner crystal domains within the fibrils become more disorganised, through mobilisation or disruption, which would then increase the remaining width average. Intracrystalline defects may also be lost through further annealing, or crystal domains may undergo further aggregation. The reference eucalyptus pulp also experienced an equivalent annealing effect with increasing hydrothermal treatment temperature, with evidence of an increase in crystallinity and a noticeable increase in apparent fibril width. No evidence of decrystallisation in the eucalyptus was observed at the highest process temperatures, although it must be remembered that this sample has already been subjected to full chemical pulping so is likely to be more stable to further processing, as evidenced by the low weight loss even at a final reactor temperature of 200°C. The comparisons suggest that hydrothermally induced decrystallisation may be exaggerated within the native lignocellulose cell wall of straw, possibly where the presence of lignin or hemicellulose breakdown products aggravate crystal ingress or disruption.

The average length of the crystal domains in the straw was possibly slightly reduced following hydrothermal treatment, according to the extrapolations from measurement of the meridional 002 and 004 reflection from the stem slivers. This may be associated with the disruption at the ends of the crystal domains of the fibrils, which are presumed to be accessible to water and hence may be plasticised or undergo hydrolytic attack. From the azimuthal scans at the 004 position, the high orientation of the epithelial fibrils was unchanged following hydrothermal treatment, from Table 1, which implies that inter-fibril organisation retains its original coherency, despite the disruption of hemicellulose and lignin fraction in the cell wall. The azimuthal scan at the 200 position suggests that hydrothermal treatment does cause slight disorientation, as the FWHH for both narrow and broad Gaussian components increased slightly. However, this may be due to the disruption of the hemicellulose and lignin fractions, which contribute to the intensity at this 2θ position. Quantification of the proportions of narrow and broad oriented components of the 200 azimuthal profiles was not attempted, as this requires full integration with respect to 2θ as well as azimuthal angle. This may be achieved in the future using 2D diffraction data.

The collective measurements show that to a large extent the size, shape and orientation distribution of the fibrils are retained in the residual cell wall structure of straw following hydrothermal treatment. The internal morphology and surface texture of the fibrils may be altered, introducing additional regions of disorder, which may influence cellulose reactivity. However, such conformational changes will be superimposed on the organisational changes due to modification of the non-cellulose matrix between fibrils and between structures in the cell wall, which will have a primary influence on fibril accessibility.

### Enzyme digestibility following hydrothermal deconstruction

Enzyme digestions of wheat straw and eucalyptus pulp were carried out up to 72 hours, to provide sufficient time points for an analysis of kinetics of glucose liberation up to high conversion. Under this regime it was considered that a semi-empirical model based on two parallel exponential type functions would be most appropriate to account for the reducing concentration of reactants (equation 8, Methods). Similar models have been employed in previous studies of enzyme hydrolysis of cellulose and biomass [5,34]. Under the fixed conditions of this study it was considered that the fitted model parameters would allow meaningful comparisons with WAXD structural information, for evaluation of possible changes in cellulose accessibility and/or reactivity induced by hydrothermal treatment. The existence of biexponential rate behaviour was interpreted as an indication of structural heterogeneity within a substrate, which could alter with processing.

**Figure 5:**
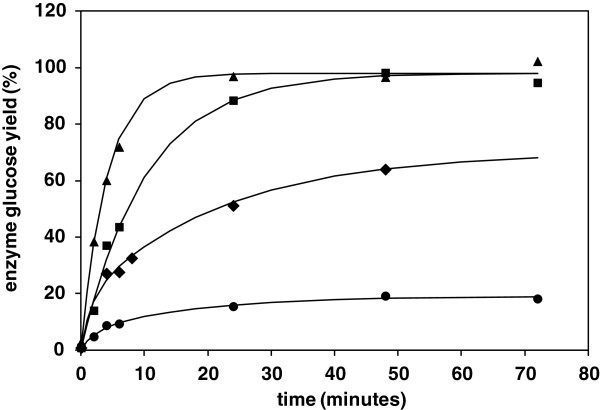
**Kinetic profiles for liberation of glucose by enzyme hydrolysis of wheat straw samples using C8546 cellulase at 40 FPU/g and 2 FPU/ml, at pH 4.9 at 50**^**o**^**C.** Continuous lines are fit to parallel exponential model with parameters shown in Table 2. Data points: (● ) untreated straw, (♦ ) straw with intermediate hydrothermal treatment up to 180^o^C, (■ ) straw with full hydrothermal treatment up to 200^o^C, (▲ ) Fully hydrothermally treated straw subjected to wet blending.

The enzyme hydrolysis profiles and resultant fits to the parallel exponential model are shown in Figure 5. Considering the as-received eucalyptus pulp, this was found to be fully digestible, but displayed biexponential rate behaviour, summarised in Table 2. Chemical refining has removed almost all lignin and hemicellulose from the pulp, which might otherwise be responsible for morphological variations. The apparent heterogeneity in the hydrolysis behaviour must therefore be due to cellulose-only factors, either reactivity differences due to local morphology along fibril strands, or accessibility differences due to variability in fibril packing. Other workers have concluded that for similar pulp materials the surface area available for direct sorption of cellulase from solution is only 4-9 m^2^/g, which is far less than the hypothetical fibril surface areas arrived at from ours and others’ calculations from WAXD fibril dimensions, between 110-170 m^2^/g [35]. Possibly not all crystal faces are equally effective for interaction, but from these surface area considerations it is most probable that enzyme access is restricted by the close packing of fibrils and the small dimensions of inter-fibrillar voids. From the kinetic model, the presence of parallel faster and slower rate constants is therefore interpreted as evidence of variations in fibril accessibility within the cell wall.

Removal of water from the inter-fibrillar voids is known to promote coalescence between adjacent cellulose surfaces through increased hydrogen bonding, which reduces the ability of the fibrils to separate on rewetting. However, even without this additional coalescence it is doubtful that the space created by removal of hemicellulose and lignin from the eucalyptus cell wall will be sufficiently large for unhindered access of cellulase enzyme. For eucalyptus, assuming an even spacing of cellulose fibrils, a hemicellulose and lignin density of 1.4 cm^3^/g, a 50:28:10 weight proportion of cellulose to hemicellulose to lignin in the original wood cell wall (estimating that 50% of total lignin is in the cell wall [6]), and a total cellulose fibril width of 4.5 nm, then a spacing of 1.5 nm is created by removal of non-cellulose material between fibrils. This is less than required for enzyme access, taking the approximate cellulase molecular dimensions of 5 nm [36]. A similar calculation for hydrothermally treated wheat straw, with a 3.7 nm total fibril width, gives 1.6 nm as the spacing created by removal of inter-fibrillar hemicellulose and lignin. The validity of these calculations could be tested in future by attempting to measure actual water volumes taken up within the cell wall, as distinct from other cellular voids. In a real straw material some expansion between fibrils may occur following hydrothermal treatment without redrying. There will be higher and lower fluctuations around the geometrical value, due to fibril aggregation, and also in the case of hydrothermally treated straw due to the presence of recondensed lignin. However, accessibility will still be limited even considering a doubling of interfibrillar dimensions, either following the crude hydrothermal processing applied to straw or the full chemical pulping applied to eucalyptus. Solute exclusion studies have shown that uncatalysed auto-hydrothermal treatment of wood leads to a moderate improvement in cell wall accessibility at the 5 nm probe size [37].

According to the analysis of enzyme hydrolysis kinetics, the cellulose in the untreated wheat straw was only 19% digestible Bi-exponential rate behaviour was observed from the model fit in Figure 5, which indicated that the straw fibrils exist in variable morphological environments. The digestibility of the straw increased with the increasing severity of hydrothermal treatment, according to 24 hour glucose yields, in Figure 4, although a sample subjected to treatment at 180°C still showed bi-exponential behaviour according to the kinetic model. The material therefore still retained its morphological heterogeneity with this intermediate severity of treatment, although the normalised average rate constant from the model was increased from 0.01 to 0.04 min^-1^ and the digestible fraction increased to 70% of total cellulose. In comparison, a sample of straw treated under optimal conditions at 200°C was 98% digestible and displayed single rate behaviour, with a faster rate constant of 0.1 min^-1^. As a general observation, the shift from bi-exponential to mono-exponential first order kinetics would seem to be coincident with the overall increase in hydrolysis rate. This implies that accessibility both increases and also becomes more uniform throughout the fibril organisation. As a further comparison, a separate digestion was carried out using straw hydrothermally treated at 200°C, which was retained in the neverdried state. A single rate constant was again observed, of 0.14 min^-1^_,_ which reflects the additional gain in accessibility in the absence of inter-fibrillar coalescence caused by drying.

Even after ideal treatment, including retention of the neverdried state, the implication of limited interfibrillar accessibility means that the complete digestion of cellulose fibrils in both pulp and wheat straw could depend on the progress of hydrolysis reactions through fibrils at the accessible surfaces of aggregates to those fibrils in underlying positions. Mechanisms including etching, pitting and particle fragmentation may therefore be important. Significantly, from Table 2, a separate WAXD comparison of part digested samples showed that for both eucalyptus pulp and hydrothermally treated straw the inherent fibril crystallinity and crystal dimensions were not significantly changed after an intermediate enzyme hydrolysis time of 6 hours, The observed biexponential rate behaviour for eucalyptus pulp is therefore not likely to be the result of faster digestion of non-crystalline cellulose. The lack of increase in fibril crystallinity during the course of digestion also suggests that cellulase enzymes have limited opportunity for selective interactions with only disordered cellulose chain segments. This is consistent with the suggested fibril structural model, where disordered polymer chains are in localised surface or defect environments rather than present as a separate amorphous phase. An interacting enzyme will therefore be in proximity of cellulose units in a range of conformations. Individual ordered and disordered chain segments may be hydrolysed at different rates but the average total rate constant may be proportional to the average conformational distribution of the whole assembly. Furthermore, it is possible that new disordered surfaces are created as enzyme etching and tunnelling progressively moves through the fibril bulk [38]. Increases in measured crystallinity following enzyme hydrolysis have been reported, sometimes based on the determination of a simple WAXD crystallinity index, although the robustness such interpretations have been questioned [17].

For straw, the measured reduction in crystallinity caused by hydrothermal treatment could lead to an improvement in the average reactivity of the fibril assembly, by increasing the average proportion of disordered chain segments. Published data for a cellulose model which was progressively decrystallised by chemical means revealed a strong inverse dependence of digestibility on crystallinity, although it is difficult to fully account for the influence of accessibility and molecular weight factors [39]. However, taking the relationship from this other study, for straw the reduction in crystallinity from 63 to 44 % following hydrothermal treatment could lead to up to a doubling of inherent fibril reactivity, based on initial hydrolysis rates. This useful additional enhancement would amplify the primary enhancement brought about by increased accessibility due to disruption of the hemicellulose and lignin components in the cell wall.

### Influence of wet state attrition

**Figure 6:**
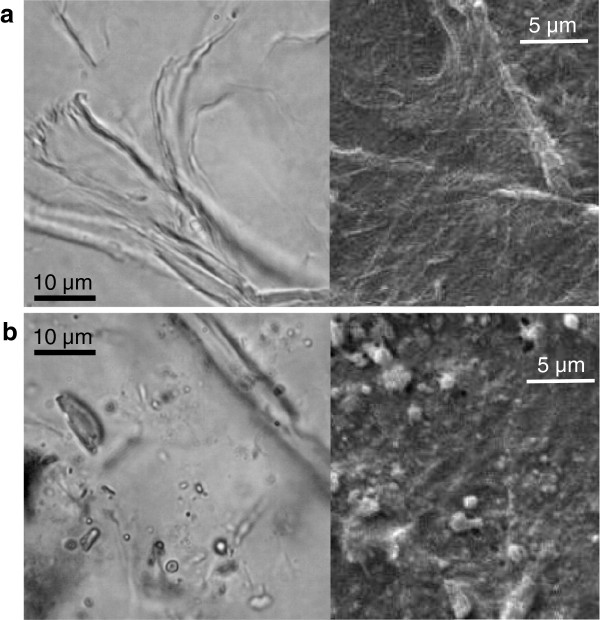
Optical microscopy (left) and scanning electron microscopy images (right) of samples subjected to high-shear wet attrition a) eucalyptus pulp showing fibrillation at fibre end b) hydrothermally treated straw, showing cell-wall fragments and lignin agglomerates, and intact vascular fibre.

The primary importance of increasing enzyme accessibility by creation of larger inter-fibrillar spaces was demonstrated by subjecting the eucalyptus pulp and hydrothermally treated straw to the process of wet attrition using a food blender. Related methods are used to improve binding between wood fibres, where small bundles of elementary fibril aggregates are peeled away from the surface of the tracheid cells. Optical microscopy and scanning electron microscopy (SEM) images of the wet-blended eucalyptus pulp are shown in Figure 6a, which was estimated to consist of 30% of the total cellulose mass in strands of separated fibril bundles, with a range of lateral dimensions with a lower limit less than 100 nm. In comparison, wet blending caused the hydrothermally treated straw to break into sub-micron fragments, rather than extended fibrils, shown in Figure 6b, probably derived from the sclerenchyma and parenchyma cells of the stem and leaf. These fragments were interspersed between more resistant lengths of vascular cells, and also spherical lignin condensates formed during hydrothermal treatment. Accurate sizing by SEM was difficult due to coalescence of the particles on drying, but typical dimensions were also estimated to be less than 500 nm, again with a lower limit of less than 100 nm. Most importantly, WAXD analysis confirmed that the crystallinity and crystal dimensions of both pulp and straw materials were unaltered by the wet blending process, so that attrition simply increased the accessible fibril surface area.

From analysis of the enzyme kinetics, the wet-blending process led to a significant increase in the average rate constant for hydrolysis of the eucalyptus pulp, from 0.03 to 0.08 min^-1^, in Table 2, which is consistent with an increase in fibril accessibility. Biexponential behaviour was still observed, with the faster rate component possibly associated with hydrolysis of the fibrils separated from the bulk fibres. The improvement in hydrolysis rate constant was equally dramatic for the hydrothermally treated straw, which increased from 0.1 to 0.24 min^-1^ following wet-blending, with almost complete digestion of cellulose achieved in 12 hours. This is again evidence that initially the fibrils in hydrothermally treated straw had incomplete accessibility, which could be enhanced by further hydro-mechanical action. The mono-exponential kinetic behaviour was apparently retained following wet-attrition, which suggests a uniform fragmentation of the straw material. As expected, the untreated straw was resistant to the wet-blending action as the intact lignocellulosic structures are sufficiently tough to withstand the hydro-mechanical forces. The deconstruction of hemicellulose and lignin by prior hydrothermal treatment weakens the adhesion between the cells and presumably also within the cell wall matrix, which enhances the effectiveness of wet-blending.

## Conclusions

The major cause of improvement in enzyme digestibility following hydrothermal deconstruction is a result of removal of hemicellulose and disruption of lignin in the cell wall, which increases accessibility of cellulase enzymes to the cellulose fibrils. Overall fibril organisation is retained following hydrothermal treatment, although crystallinity is reduced, which may enhance local enzyme reactivity at fibril surfaces. However, space created between fibrils by disruption of hemicellulose and lignin is believed to be insufficient for complete penetration by cellulases, so the observed total digestion of hydrothermally treated cellulose may require etching type mechanisms. Significant additional improvement in enzyme hydrolysis rates could be achieved by increasing accessibility, by inducing the separation of fibril aggregates by wet attrition, which may be of commercial application. From WAXD analysis, the lack of preferential digestion of non-crystalline cellulose is consistent with the presence of local conformational disorder at fibril surfaces and defects, rather than separate amorphous cellulose domains. If this interpretation is correct then the small length scale of disorder suggests that cellulase interactions with fibril surfaces may not be fully specific towards particular conformational states. Improvements in enzyme reactivity caused by reduction in total crystallinity may operate on an assembly average basis.

## Methods

### Samples

Wheat straw (Zebedee variety) was harvested from the University of Nottingham farm, which was stored under dry ambient conditions prior to investigations. The complete proportions of stem and leaf components were knife milled to a 2 mm mesh size (Pulverisette 19. Fritsch Gmbh), to form a homogeneous stock suitable for hydrothermal treatments. A further size reduction of milled samples was carried out prior to powder-type WAXD measurements, to 0.5 mm mesh size, using a laboratory hammer mill (Perten Gmbh). Hydrothermal treatments were also carried out on complete 2-3 cm lengths of stem internodes, for preparation of samples for fibre type WAXD measurements. From previous work the gross composition of this straw feed stock was: cellulose 39%, hemicelluose 28%, lignin 20%, by weight [24]. Comparative investigations were also carried out on a standard eucalyptus dissolving pulp, consisting of 96% α-cellulose and 4% hemicellulose (xylan), with minimal lignin (supplied by Lenzing AG), which was fluffed using a domestic coffee grinder. This form was used for hydrothermal treatments and was also suitable for powder WAXD measurements.

### Hydrothermal treatments

2 gram portions of 2 mm mesh size straw were mixed with 8 mls of demineralised water and sealed into 1 inch diameter stainless-steel tube reactors, which were then placed for different times in an air-circulation oven set at a temperature of 200°C [24]. The time in the oven was noted from the point of insertion, with the reactor temperature following a ballistic heating profile, from ambient to the oven air temperature, as measured separately by a thermocouple probe. From this known profile it was possible to estimate the final inner reactor temperature for each time interval. After removal from the oven, the tubes were quickly cooled under running cold water and the contents were then vacuum-filtered to separate the treated solid from the hydrolysate liquor. The solid was washed with repeated amounts of demineralised water (6 × 200 ml) and the filter paper and treated product were then air-dried overnight, then dried for a further two hours at 100°C, then cooled and reweighed to provide a measure of mass loss. The ambient moisture content of the untreated straw was determined separately, for accurate mass calculations. Equivalent experimental procedures were used to treat whole stem samples for selected times, with sufficient liquor added to the reactor tube to ensure full immersion of the stem lengths. Samples of fluffed eucalyptus pulp were also subjected to hydrothermal treatment at selected temperatures. Drying is known to have an impact on subsequent enzyme accessibility but was a necessary preparation for analytical measurements following hydrothermal treatment [40]. Under the same drying conditions it was considered that all comparisons between straw samples would be valid. For completeness, a separate neverdried hydrothermally treated sample of straw was also prepared, to establish the typical effect of drying on enzyme hydrolysis rate.

### Wet state attrition

Water suspensions of eucalyptus pulp and hydrothermally treated straw were subjected to extended wet attrition using a domestic food blender (Kenwood Ltd). 3 gram amounts of dry material were blended in 500 mls of water, on fast setting, under timed control with a cycle of 2 minutes on, 1 minute off, with additional pauses of 10 minutes after every 20 minutes running, to avoid undue sample heating. The total blending time for each sample was 240 minutes. Following treatment, the suspensions were diluted directly with a concentrate of citrate buffer and a stock of predialised enzyme concentrate, to make up a final suspension for digestion to determine hydrolysis kinetics, as outlined below.

### X-ray diffraction

#### Powder measurements

0.5 mm mesh size samples of untreated and treated straw were measured in reflection mode, with a vertical θ-θ Bragg-Brentano diffractometer (D5000, Siemens / Bruker Gmbh). Irradiation was from a copper Kα X-ray source (λ = 1.541 Angstroms), with static slit optics at source and detector. Samples were loaded into circulator holders with light pressure, which were rotated about the phi-axis during measurement. 2θ scans were performed from 5 to 50°, in 0.05° increments. Preliminary investigations highlighted the natural preferential alignment of larger fibrous particles in the sample plane, which was minimised by the selected size reduction conditions. These conditions gave random shaped particles with low aspect ratio, with the minimum input of mechanical energy, ensuring retention of plant cell wall structures, as confirmed by optical microscopy. This method avoids further manipulation of the samples, for example, by pressing or wetting and drying, although it is recognised that some residual particle alignment may be retained, which may slightly influence absolute crystallinity values.

Intensities were background corrected by linear subtraction between 5 and 50° 2θ, which provided an approximate correction for air, instrumental and incoherent contributions to scattering. Polarisation correction was performed over this 2θ range. Absorption corrections were not deemed necessary for the reflection geometry with these low absorbing materials, with thermal diffuse scattering considered to contribute as a constant factor [16]. Separation of crystalline and non-crystalline diffracted intensity was carried out by subtraction of experimental amorphous profiles from the total coherent profiles, obtained by extended ball-milling of each specific sample, of both untreated and hydrothermally treated straw and eucalyptus pulp. A single amorphous scale factor was determined by allowing the amorphous profile to just touch the total profiles at a value around 19° 2θ, which is considered to be a position of minimal crystalline intensity.

Total sample crystallinity was calculated by integration of the invariant between 5 and 50° 2θ using equation (3), where the angular axis of the diffraction curves was changed from 2θ to s units, where s is the scattering vector = 2sinθ/λ, and I_cr_ and I_tot_ are crystalline and total intensities. The constant K (equation 4) is a correction factor for crystal defects, where ${\bar{f}}^{2}$ is taken as the mean square atomic scattering factor for cellulose [21]. D is a disorder function defined as D = exp(-ks^2^), with k a damping constant taken from literature values for cellulose.

(3) $$\mathrm{Cr}\left(\%\right)=100\times \frac{\;\int_{s0}^{s1}I_{\mathrm{cr}}{\left(s\right)s}^{2}\mathrm{ds}}{\int_{s0}^{s1}I_{\mathrm{tot}}\left(s\right)s^{2}\mathrm{ds}}.K\;$$

(4) $$K=\frac{\int_{s0}^{s1}s^{2}{\bar{f}}^{2}\mathrm{ds}}{\int_{s0}^{s1}s^{2}{\bar{f}}^{2}\mathrm{Dds}}$$

#### Fibre measurements

Fibre type measurements were carried out in transmission mode, using a D8 Discover horizontal goiniometer system (Bruker Gmbh), fitted with a quarter circle Eulerian cradle, with copper Kα X-ray source, with parallel spot optics and 1 mm source and detector apertures. Individual longitudinal slivers of stem or leaf samples were clamped in a circular holder, mounted at the geometrical axis. Meridional θ-2θ scans were performed from 5-50°, in the plane parallel to the stem axis, with azimuthal scans carried out by rotation in the sample plane, with concatenation as required, with source and detector fixed at the 2θ position of the 200 and 004 cellulose-I reflections. Duplicate meridional and azimuthal measurements were performed to confirm reproducibility of results, with an individual data set used for analysis. Linear background subtraction of the meridional 002 and 004 reflections were performed between points of minimum intensity immediately on either side of the strong peak intensity. Background corrections to azimuthal profiles were carried by horizontal linear subtraction of a separately determined blank background, with awareness of tails of off-axis reflections. Assuming uniaxial symmetry, a measure of the extent of fibril disorientation with respect to the stem axis was found by calculation of the full width at half height (FWHH) of the 200 and 004 azimuthal profiles, after fitting to single or superimposed Gaussian function(s). The average angle of disorientation ($\bar{\varphi }\;$) was calculated using equation (5), where I is the intensity at the azimuthal angle (φ) [29].

(5) $$\bar{\cos^{2}\varphi }=\frac{\int_{0}^{\pi /2}\;I\left(\varphi \right){.\cos}^{2}\varphi .\sin^{2}\varphi \;\mathrm{d\varphi}}{\int_{0}^{\pi /2}\;I\left(\varphi \right).\sin^{2}\varphi \;\mathrm{d\varphi}}$$

### Crystal dimensions

After amorphous subtraction of the powder diffractograms, the remaining crystalline intensity of original and treated straw samples could be fitted successfully to a simulation consisting of four Gaussian curves, representing the major reflections of cellulose-I, at 2θ = 14.7° (1-10), 16.6° (110), 20.9° (102) and 22.5° (200) [41]. The choice of Gaussian shapes for straw resulted in the most effective fitting of the strong 200 reflection, in conjunction with the amorphous subtraction procedure. A non-linear least-squares minimisation was carried out using the Solver tool add-in within Microsoft Excel^(^TM^)^, with intensity and width allowed to float freely from initial manual estimates, but positions fixed within +/- 1° limits. As discussed, although fitting of the overlapped 1-10 and 110 peaks was successful using a Gaussian function, this was not possible with a unique solution. The position of the 102 reflection was fixed and its intensity was floated with its breadth equal to and its intensity not exceeding 15% of the 200 reflection. An equivalent procedure was used for the eucalyptus pulp samples, but here the four sharper crystalline peaks were best fitted to pseudo-Voigt (Gaussian + Lorentzian) shapes, although still with a strong Gaussian component. The choice of this mixed function was empirical and for simplicity in the mathematical simulation, as it is appreciated that other complex peak shapes are also considered for cellulose. However, the value of integral breadth as used for dimensional calculations is not dependent on the mathematical peak form. The reflections at 2θ = 17.2° (002) and 2θ = 34.9° (004), measured from the meridional scans of the straw stems, were also fitted most effectively to pure Gaussian functions, with the deconvolution of the 004 peak based on the sum of two Gaussians where appropriate. Deconvolution of the 002 peak was not attempted and it was estimated that any broader feature would be removed by the background subtraction.

The crystal dimensions associated with the different X-ray reflections were calculated from diffraction peak widths according to the Scherrer method, with the constant 0.9 selected for consistency with published studies [26]. The sharper 002 and 004 meridional peaks were corrected for the effect of instrumental broadening, according to equation (6), where the integral breath IB = peak height/peak area, and IB_exp_ and IB_std_ respectively are the integral breadths of the fitted peak and the diffraction standard. The integral breadth of a polycrystalline silicon standard was taken as 0.148°. A further correction for the effect of defect broadening was applied for calculation of the average crystal length, by determining IB_size_ from the intercept of the plot of IB^2^ vs m^4^ for the 002 and 004 peaks [27]. Corrections for instrumental and defect broadening were not deemed necessary for the broad 200 peak. Crystal dimensions (d) were calculated using equation (7), although for the straw powder diffactograms the lower intensity and overlap of the 1-10, 110 and 102 reflections meant that only the stronger 200 reflection was suitable for comparisons.

(6) $$\mathrm{IB}=\sqrt{{\left(IB_{\exp}\right)}^{2}-{\left(IB_{\mathrm{std}}\right)}^{2}}$$

(7) $$d=\frac{0.9.\lambda }{\mathrm{IB}.\cos\left(\theta \right)}$$

#### Enzyme hydrolysis kinetics

Dry 200 mg portions of untreated or treated straw material were mixed with enzyme in 40 mls of sodium citrate buffer (pH 4.9) in 100 ml screw-cap falcon tubes, giving a 0.5% solids concentration. A commercial Trichoderma reesei cellulase formulation was used (C8546 lyophilised powder, ex Sigma Aldrich Co Ltd), which was applied at high enzyme to substrate ratio and low solids concentration (40 FPU/g biomass, 2 FPU/ml). These conditions were aimed at achieving saturation of sorption sites, whilst minimising build-up of intermediate or end products, which might otherwise lead to enzyme inhibition at extended reaction times. A preconcentrate of the enzyme was dialised overnight to remove extraneous sugars, under conditions which avoided degradation of the membrane material. Digestions were carried out at 50°C on an orbital shaker at 150 rpm. Up to 7 sample aliquots each of 250 μl volume were removed at increasing time intervals, which was an amount considered to have insignificant effect on the liquor-solid ratio during the digestion. The glucose monomers in each aliquot were determined by high-performance anion exchange chromatography with pulsed amperometric detection (Dionex, UK), using a CarboPac PA20 column with isocratic system at working flow rate of 0.5 ml/min. Quantification was performed using calibration curves from glucose standard solutions, with mannitol as an internal standard. Fractional yields were calculated by reference to the total glucose liberated by full acid hydrolysis of the straw materials in 12M sulphuric acid for 1 hour at 30°C, followed by 2 hours in 1M sulphuric acid at 98°C.

Experimental values for glucose release were first expressed as a fraction of the theoretical 100% glucose yield possible for each sample. A non-linear optimisation method was used to fit a simulated curve to the experimental points, describing the sum of two first-order exponential processes, according to equation (8), where S is the glucose yield at time t, A and B are the proportions of the two processes and x and y are the respective time constants. The sum A+B was not allowed to exceed 100%, but could be less, with the shortfall representing a non-digestible fraction. Yield data at 24 hours was also used to follow trends hydrothermal treatment temperature.

(8) $$S=A\left(1-e^{-\mathrm{xt}}\right)+B\left(1-e^{-\mathrm{yt}}\right)$$

For comparisons, the rate constants were also normalised as a proportion of the contribution of the particular component to the theoretical 100% yield. Additionally, the biexponential rate constants were averaged, for comparisons with single rate behaviour.

#### Optical and scanning electron microscopy

A Hitachi S300 instrument was used under high vacuum mode. Samples were coated by gold sputtering and images were acquired typically at 14 kV accelerating voltage. Drops of diluted wet suspensions were allowed to dry directly onto the SEM stub, left overnight at ambient temperature. Optical micrographs were obtained using water as the dispersing medium.

## Abbreviations

WAXD: Wide angle X-ray diffraction; FWHH: Full width at half height; SEM: Scanning electron microscopy.

## Competing interests

The authors declare that they have no competing interests

## Author’s contributions

Physical treatments and structural characterisation carried out by RI, Chemical analysis and enzymatic studies carried out by SG, Cell wall structures and models developed with support of GT, Pretreatment technologies developed with support of SH. All authors read and approved the final manuscript.
